# Fondaparinux-associated Thrombocytopenia

**DOI:** 10.12669/pjms.37.2.3522

**Published:** 2021

**Authors:** Jahanzeb Malik, Nismat Javed, Matiullah Kamin

**Affiliations:** 1 Dr. Jahanzeb Malik, Rawalpindi Institute of Cardiology, Rawalpindi, Pakistan; 2 Dr. Nismat Javed, Shifa College of Medicine, Shifa Tameer-e-Millat University, Islamabad, Pakistan; 3 Dr. Matiullah Kamin, Shifa International Hospital, Islamabad, Pakistan

**Keywords:** Factor Xa inhibitor, Fondaparinux-associated thrombocytopenia, HIT

## Abstract

Heparin-induced thrombocytopenia (HIT) is an immune-mediated condition causing thrombocytopenia and paradoxical thrombosis after exposure to heparin or low-molecular-weight heparin. It has been rarely reported by Fondaparinux, an artificial pentasaccharide similar to heparin. This manuscript presents a case of HIT associated with fondaparinux use.

## INTRODUCTION

Heparin-induced thrombocytopenia (HIT) is a prothrombotic condition associated with heparin exposure.^1^ The immunoglobulin G antibodies, which clump heparin molecule and platelet factor 4, causes the clinical syndrome of thrombocytopenia and thrombo-occlusive manifestations.^2^ HIT typically occurs after 5 to 10 days of exposure to heparin.^2^ This syndrome is most commonly associated with unfractionated heparin while it is described uncommonly with low-molecular-weight heparin.^3^,^4^ Fondaparinux is a synthetic pentasaccharide that inhibits factor Xa by binding to antithrombin III.^4^ In low-risk Non-ST elevation myocardial infarction (NSTEMI), it is as effective as unfractionated heparin or low-molecular-weight heparin.^5^ Due to the mechanism of action, Fondaparinux is not able to bind the platelet Factor-4 to generate immune complexes leading to platelet activation. The risk of HIT is thought to be negligible with Fondaparinux, proposing this as a safe drug in the treatment of HIT itself, along with other indications.^6^ We describe a case of HIT associated with fondaparinux in a patient with acute coronary syndrome.

### Patient and observation

A 46 years-old male presented with complaints of central on-going chest pain which started a week ago. Taking it as acid peptic disease he did not consult any medical personnel until it worsened since yesterday. He was diagnosed as NSTEMI based on ECG and Troponin-I essay. His comorbid included a family history of ischemic heart disease and smoking. His thrombolysis in myocardial infarction (TIMI) score was two and the Global Registry of Acute Coronary Events (GRACE) score was 102. Guideline directed medical therapy (GDMT) was started. He was given Fondaparinux 2.5 mg once daily till hospitalization. At baseline, his platelets were 256,000/µL. On the second day, platelets dropped to 110,000/µL. They were repeated on the suspicion of lab error, which showed 90,000/µL. His history was not significant for dengue or associated infections and thrombocytopenia. His platelet Factor-4 was positive. Autoimmune profile and viral serology were normal. After ruling out the common causes of thrombocytopenia, the diagnosis of HIT was made. Fondaparinux was discontinued and intravenous infusion of lepirudin was started. The next day platelet count increased to 148,000/µL. The following day, the count was 205,000/µL. After three days of lepirudin intravenous infusion, it was discontinued and the patient was discharged on clopidogrel 75 mg with a platelet count of 223,000/µL. On follow-up after two weeks his platelets were 220,000/µL.

## DISCUSSION

Thrombocytopenia associated with heparin is a common complication seen in every day clinical practice. Treatment of HIT constitutes discontinuing heparin and substituting direct thrombin inhibitors (DTIs).^7^ Lepirudin and Argatroban are the commonly used DTIs in HIT. Newer DTI include bivalirudin. Fondaparinux, another DTI, has also been used in HIT with successful results.^7^ In our case, however, fondaparinux was involved in causing HIT after the first dose. The platelets decreased by approximately 50%. After discontinuing Fondaparinux platelet count increased to normal value. This indicates a role of Fondaparinux in causing HIT in sporadic cases. On literature review, eight cases of thrombocytopenia with this factor Xa inhibitor since 2007 have been reported.^8^-^10^

## CONCLUSION

Despite many reports of successful treatment of HIT with fondaparinux, there seems to be a possibility of fondaparinux causing HIT, as suggested by our case report. Further trials are necessary to clarify the mechanism behind fondaparinux-associated thrombocytopenia.

### Learning Points:

- Heparin-induced thrombocytopenia is fairly common but Fondaparinux causing decreased platelets is not seen regularly.
- In the literature, Fondaparinux has been used successfully for the treatment of heparin-induced thrombocytopenia in addition to conventional direct thrombin inhibitors.
- Our case shows one of the nine reports in the literature since the first presentation of Fondaparinux-associated thrombocytopenia in 2007.

### Author’s Contributions:

**JM:** Idea, design, and editing of final draft. He takes the responsibility and is accountable for all aspects of the work in ensuring that questions related to the accuracy or integrity of any part of the work are appropriately investigated and resolved.

**NJ:** Case selection and manuscript writing.

**MK:** Manuscript writing and supervision.
